# Could the Polymorphisms of DOCK4 (rs147636134), SYNGAP1 (rs199759879), and FOXP1 (rs767001715) be the Primary Risk Factors for Bipolar Disorder and Autism Spectrum Disorder?

**DOI:** 10.1002/dneu.22995

**Published:** 2025-08-11

**Authors:** Elvan Çiftçi, Nimet Sağlam, Tayfun Gözler, İpek Yüksel, Neriman Kilit, İlknur Bozkurt, Muhsin Konuk, Korkut Ulucan, Nevzat Tarhan

**Affiliations:** ^1^ Department of Psychiatry NPI Brain Hospital, Uskudar University Istanbul Turkey; ^2^ Department of Neuroscience Uskudar University Istanbul Turkey; ^3^ Department of Child and Adolescent Psychiatry NPI Brain Hospital, Uskudar University Istanbul Turkey; ^4^ Department of Biochemistry Uskudar University Istanbul Turkey; ^5^ Department of Molecular Biology and Genetics Uskudar University Istanbul Turkey

**Keywords:** autism spectrum disorder, bipolar disorder, Dedicator of Cytokinesis 4 (DOCK4), Forkhead Box P1 (FOXP1), neurodevelopmental disorder, neuropsychiatric disorder, single nucleotide polymorphism, Synaptic Ras GTPase‐activating protein 1 (SYNGAP1)

## Abstract

Autism spectrum disorder (ASD) and bipolar disorder (BD) are psychiatric diseases that may overlap in common neurodevelopmental and genetic basis. Forkhead Box P1 (FOXP1), Synaptic Ras GTPase‐activating protein 1 (SYNGAP1), and Dedicator of Cytokinesis 4 (DOCK4) genes are critical for synaptic plasticity, neuronal communication, and brain development. This study aims to investigate the association of *FOXP1* (rs767001715), *SYNGAP1* (rs199759879), and *DOCK4* (rs147636134) polymorphisms with ASD and BD and to determine the effects of genetic variations on disease pathogenesis in the Turkish population. This study was conducted with a total of 200 participants, including 50 ASD patients, 50 BD patients, and 100 healthy controls. DNA was isolated from peripheral blood samples, and *FOXP1*, *SYNGAP1*, and *DOCK4* polymorphisms were genotyped using real‐time PCR. The distribution of genetic variants was compared between patient groups and healthy controls. The chi‐square test was applied for statistical analyses. In terms of *FOXP1* (rs767001715), *SYNGAP1* (rs199759879), and *DOCK4* (rs147636134) polymorphisms examined in the study, no statistically significant difference was found between the ASD and BD patient groups and the healthy control group (*p* > 0.05) in the Turkish population. In addition, it was determined that these variants had allele frequencies compatible with global population data. However, due to the limited sample size, these results cannot be generalized. Further large‐scale population analyses and functional studies are needed to investigate the association of these genes with ASD and BD in more detail.

## 1 Introduction

Autism spectrum disorder (ASD) and bipolar disorder (BD) are heterogeneous conditions with complex genetic etiologies. While the genetic basis of ASD is relatively well characterized, BD remains less understood. Recent research has explored overlaps between neurodevelopmental disorders (e.g., ASD) and functional psychiatric disorders (e.g., BD), particularly in shared synaptic dysfunction and risk genes (Masini et al. 2020). Both common and unique gene‐expression perturbation patterns are found across neuropsychiatric disorder. Interestingly, polygenic (single‐nucleotide polymorphism [SNP]‐based) overlap across disorders is correlated with the degree of transcriptional dysregulation sharing, indicating a substantial genetic component at play. Disrupted synaptic processes and astrocyte activation are hallmarks of the global gene expression patterns shared by BD and autism (Gandal et al. 2018).

The largest exome sequencing study of ASD has identified 102 risk genes, with 49 showing higher frequencies of disruptive de nova variants with severe neurodevelopmental delay and 53 in those with ASD. These genes regulate gene expression and neuronal communication, and their expression is enriched in excitatory and inhibitory neuronal lineages, indicating multiple pathways to ASD (Satterstrom et al. 2020). On the other hand, the heritability of bipolar disorder, which is thought to be between 60% and 80%, has been amply demonstrated by twin and adoption studies. This imperfect concordance rate suggests that gene × environment interactions play a key role in the risk and onset of the disorder. Recent genome‐wide association studies have identified susceptibility genes for BD. BD is a complex polygenic disorder caused by the combination of multiple common variants, individually of small effect, that account for ∼30% of the heritability seen in bipolar disorder (Walss‐Bass 2021).

Genes such as Forkhead Box P1 (FOXP1)**, **Synaptic Ras GTPase‐activating protein 1 (SYNGAP1), and Dedicator of Cytokinesis 4 (DOCK4) play critical roles in synaptic plasticity, neuronal communication, and brain development. Investigating these genes may uncover common pathogenic mechanisms between ASD and BD. Common alleles are typically older compared to rare alleles. The complex evolution of humans, including different adaptations, admixtures, and migration events, is reflected in these common SNPs that first emerged thousands of years ago (Fedorova et al. 2022).

FOXP1 is strongly associated with neurodevelopmental impairments, including ASD and cognitive dysfunction (Chien et al. 2013). As a transcriptional regulator, it influences cortical gene expression and synaptic plasticity (Araujo 2017). *FOXP1* has been identified as a high‐confidence risk factor for ASD, and mutations in this gene have been shown to lead to neurodevelopmental disorders by affecting language and cognitive functions (Nisar et al. 2022). Mutations in *FOXP1* are linked to language deficits, intellectual disability, and social communication deficits (Bacon and Rappold 2012). Notably, *FOXP1* overexpression has been observed in ASD, potentially disrupting neuronal connectivity (Chien et al. 2013). Beyond ASD, dysregulated *FOXP1* expression has been implicated in BD and schizophrenia (Guan et al. 2019), suggesting its role in synaptic gene regulation and neurotransmitter systems may contribute to shared phenotypes in both disorders (Castro Martínez et al. 2019).

SYNGAP1, a key regulator of synaptic function, is implicated in ASD and other neuropsychiatric conditions (Birtele et al. 2022). *SYNGAP1* encodes a postsynaptic protein that regulates neuronal plasticity and synaptic connections. Haploinsufficiency of *SYNGAP1* is associated with epilepsy, intellectual disability, and ASD‐like traits (Xing et al. 2016). Its role in glutamatergic signaling and synaptic plasticity suggests a potential link to BD, where disrupted NMDA receptor‐mediated signaling may underlie cognitive and mood dysregulation (Birtele et al. 2022; Kalkman 2012). These findings highlight the important role of *SYNGAP1* in the shared neurodevelopmental mechanisms underlying diseases such as ASD and BD.

DOCK4, a guanine nucleotide exchange factor (GEF) for Rac1, is associated with neuropsychiatric diseases. Dock4 plays a crucial part in neurite differentiation in the early stages of neuronal development (Xiao et al. 2013). It influences synaptic plasticity, neuronal morphogenesis, and cellular signal transduction. Genetic studies associate *DOCK4* mutations with ASD (Maestrini et al. 2010; Nisar et al. 2022), and specific SNPs may increase risk (Bailur et al. 2019). Regarding BD, the role of DOCK4 remains less well defined; however, studies suggest a potential link between this gene and BD. *DOCK4* has been shown to play a role in shaping neuronal circuits and is particularly expressed in regions such as the hippocampus, which may be associated with neurodevelopmental disorders seen in BD (Akahoshi and Yamamoto 2018), and microdeletions in *DOCK4* have been proposed to contribute to the cognitive and behavioral deficits observed in both ASD and BD (Bloch‐Gallego and Anderson 2023). Mechanistically, *DOCK4* may modulate neuroplasticity via Rho GTPase pathways (Shi 2013).

These genetic insights could advance personalized diagnosis and treatment for ASD and BD. Pharmacogenetic studies, in particular, may identify novel therapeutic targets by examining shared or disorder‐specific risk variants. This study aims to analyze these three polymorphisms for the first time in a Turkiye cohort. Three single nucleotide polymorphisms in *FOXP1*, *SYNGAP1*, and *DOCK4* are analyzed to be able to evaluate genetic predisposition and correlate variations with clinical findings of ASD and BD compared to healthy control subjects.

## 2 Material Method

### 2.1 Participants

The participants in this study consist of individuals diagnosed with ASD and BD based on the DSM‐5 (Diagnostic and Statistical Manual of Mental Disorders, Fifth Edition) criteria, as well as healthy controls. The participants were recruited between 2021 and 2024 from NP Istanbul Brain Hospital. The patient study group includes a total of 100 patients comprising 50 ASD and 50 BD patients. Additionally, a total of 100 healthy individuals without any neuropsychiatric disorder diagnosis were included in the study. Thus, analyses were conducted on a total of 200 participants.

Assessing the genotype–phenotype relationship in ASD and BD is a crucial step in understanding the genetic basis of these disorders. All participants provided written informed consent, were given detailed information about the study, and had the opportunity to ask questions.

To diagnose ASD and BD according to DSM‐5 criteria, all participants were evaluated with the DSM‐5 Structured Clinical Interview (SCID‐5) method and went through semi‐structured clinical interviews (Hamdan et al. 2010).

This study was approved by the Üsküdar University Ethics Committee and was conducted in accordance with the Helsinki Declaration (Ethics Committee Approval Number: 61351342/2020‐169, date: 27.03.2020).

### 2.2 Collection of Blood Samples From Patients and Control Groups

In this study, blood samples were collected from patients diagnosed with ASD and BD and healthy control subjects without any psychiatric diagnosis for routine biochemical tests that did not require intervention. Venous blood samples were collected in tubes containing EDTA as an anticoagulant. Samples were stored at +4°C until transported to the laboratory, and if DNA isolation could not be performed on the same day, they were stored at −20°C. Samples requiring long‐term storage were stored at −80°C to preserve DNA integrity. All samples were stored securely in the laboratory and converted to code numbers to ensure the confidentiality of participant information. Only the research coordinator had access to this information, thus preventing disclosure of volunteer names or possible ethical issues.

### 2.3 Genotyping Analysis

DNA isolation from peripheral blood samples was performed using the commercially available PureLink Genomic DNA isolation kit (Invitrogen, Van Allen Way, Carlsbad, CA, USA) according to the manufacturer's instructions. Analysis of *FOXP1* (rs767001715), *SYNGAP1* (rs199759879), and *DOCK4* (rs147636134) polymorphisms was performed using the TaqMan Genotyping Assays (Applied Biosystems, Foster City, CA, USA) genotyping kit according to the manufacturer's instructions using the Thermo Fisher Quanti Studio 5 Real‐Time PCR (Thermo Scientific, Waltham, Massachusetts, USA) instrument. The TaqMan genotyping method was preferred because it provides rapid and highly sensitive detection of target SNPs. To increase experimental accuracy, 10% of the samples were randomly selected and genotyped twice, and the results were confirmed to be consistent. In addition, the risk of contamination was evaluated, and the absence of DNA extraction and PCR contamination was confirmed using negative controls.

### 2.4 Statistical Analysis

Statistical analysis of the data obtained from genotyping results was performed using IBM SPSS Statistics for Windows, Version 25.0 (IBM Corp., Armonk, NY, USA) software. Numerical variables are presented as mean ± SD, while categorical variables are shown as frequency (*n*) and percentage (%). Differences between age and genotype groups were evaluated using an independent *t*‐test, and the relationship between categorical variables was analyzed using Pearson's chi‐square test and Fisher's exact test. A *p* value of less than 0.05 (*p* < 0.05) was considered statistically significant.

## 3 Results

As seen in Table 1, no significant difference was found between the patient and control groups in terms of age (*p* = 0.790) and gender (*p* = 0.474).

**TABLE 1:** Comparison of age and gender between patient and control groups.

	Healthy control subjects (*n* = 100)	Patients (*n* = 100)	*p*
Age, mean ± SD	30.41 ± 10.35	29.95 ± 11.93	0.790^a^
Sex, *n*(%)			
Male	60 (60.0)	55 (55.0)	0.474^b^
Female	40 (40.0)	45 (45.0)	

^a^ Independent *t*‐test, *p* < 0.05 is statistically significant.

^b^ Pearson chi‐square test.

As seen in Table 2, genotype and allele distributions of *FOXP1* (rs767001715), *SYNGAP1* (rs199759879), and *DOCK4* (rs147636134) polymorphisms were compared between BD patients and the healthy control comparison group. Regarding the *FOXP1* (rs767001715) polymorphism, all BD patients and healthy controls had the GG genotype. The AG and AA genotypes were not detected in any individual. In terms of allele distribution, the G allele was observed at a 100% frequency, while the A allele was absent in both groups. The difference between the groups was not statistically significant (*p* = 1.000). For the *SYNGAP1* (rs199759879) polymorphism, all individuals in both the BD patient group and the healthy control group had the CC genotype. CT and TT genotypes were not observed. In allele frequency analysis, the C allele was found at a 100% frequency in both groups, while the T allele was absent. The difference between the groups was not statistically significant (*p* = 1.000). In terms of the *DOCK4* (rs147636134) polymorphism, all BD patients and healthy controls had the AA genotype, with no AG or GG genotypes observed. Regarding allele distribution, the A allele was present at a 100% frequency in both groups, while the G allele was absent. The difference between the groups was not statistically significant (*p* = 1.000).

**TABLE 2:** Genotype and allele distributions of BD patients.

*FOXP1* (rs767001715) Genotype	BD N (%)	Healthy control N (%)	*p*
GG	50 (100.0)	100 (100.0)	1.000
AG	0 (0.0)	0 (0.0)	
AA	0 (0.0)	0 (0.0)	
Allele			
G	100 (100.0)	100 (100.0)	1.000
A	0 (0.0)	0 (0.0)	
*SYNGAP1* (rs199759879) Genotype			
CC	50 (100.0)	100 (100.0)	1.000
CT	0 (0.0)	0 (0.0)	
TT	0 (0.0)	0 (0.0)	
Allele			
C	100 (100.0)	100 (100.0)	1.000
T	0 (0.0)	0 (0.0)	
*DOCK4* (rs147636134) Genotype			
AA	50 (100.0)	100 (100.0)	1.000
AG	0 (0.0)	0 (0.0)	
GG	0 (0.0)	0 (0.0)	
Allele			
A	100 (100.0)	100 (100.0)	1.000
G	0 (0.0)	0 (0.0)	

*Note*: Fisher's exact test, *p* < 0.05 is statistically significant.

As seen in Table 3, genotype and allele distributions of *FOXP1* (rs767001715), *SYNGAP1* (rs199759879), and *DOCK4* (rs147636134) polymorphisms were compared between ASD patients and the healthy control group. Regarding the *FOXP1* (rs767001715) polymorphism, all ASD patients and healthy controls had the GG genotype. The AG and AA genotypes were not detected in any individual. In terms of allele distribution, the G allele was observed at a 100% frequency, while the A allele was absent in both groups. The difference between the groups was not statistically significant (*p* = 1.000). For the *SYNGAP1* (rs199759879) polymorphism, all individuals in both the ASD patient group and the healthy control group had the CC genotype. CT and TT genotypes were not observed. In allele frequency analysis, the C allele was found at a 100% frequency in both groups, while the T allele was absent. The difference between the groups was not statistically significant (*p* = 1.000). In terms of the *DOCK4* (rs147636134) polymorphism, all ASD patients and healthy controls had the AA genotype, with no AG or GG genotypes observed. Regarding allele distribution, the A allele was present at a 100% frequency in both groups, while the G allele was absent. The difference between the groups was not statistically significant (*p* = 1.000).

**TABLE 3:** Genotype and allele distributions of ASD patients.

*FOXP1* (rs767001715)	ASD N (%)	Healthy control N (%)	*p*
Genotype			
GG	50 (100.0)	100 (100.0)	1.000
AG	0 (0.0)	0 (0.0)	
AA	0 (0.0)	0 (0.0)	
Allele			
G	100 (100.0)	100 (100.0)	1.000
A	0 (0.0)	0 (0.0)	
*SYNGAP1* (rs199759879) Genotype			
CC	50 (100.0)	100 (100.0)	1.000
CT	0 (0.0)	0 (0.0)	
TT	0 (0.0)	0 (0.0)	
Allele			
C	100 (100.0)	100 (100.0)	1.000
T	0 (0.0)	0 (0.0)	
*DOCK4* (rs147636134) Genotype			
AA	50 (100.0)	100 (100.0)	1.000
AG	0 (0.0)	0 (0.0)	
GG	0 (0.0)	0 (0.0)	
Allele			
A	100 (100.0)	100 (100.0)	1.000
G	0 (0.0)	0 (0.0)	

*Note*: Fisher's exact test, *p* < 0.05 is statistically significant.

## 4 Discussion

Psychiatric disorders such as ASD and BD are highly polygenic, with numerous genetic loci each contributing small effects (Wray et al. 2018). While genomic studies have elucidated the complex genetic architecture of these disorders, the role of specific SNPs and candidate genes remains incompletely understood. In this study, we investigated the potential association of FOXP1 (rs767001715), SYNGAP1 (rs199759879), and DOCK4 (rs147636134) polymorphisms with ASD and BD in a Turkish cohort. Our findings revealed no statistically significant differences in these variants between patient groups and healthy controls. However, these results should be interpreted cautiously, as they are limited by sample size and the specific SNPs examined. Further studies with larger cohorts and broader genetic analyses are needed to conclusively determine whether these genes contribute to ASD and BD pathogenesis.

### 4.1 Genetic Basis of ASD Versus BD

Both common and uncommon variants make up the genetic landscape of ASD, which can differ from person to person (Bourgeron 2015). Large‐scale sequencing has also revealed a number of high‐confidence risk genes for ASD, indicating that the disorder has a well‐established genetic basis. In contrast, BD exhibits a more complex polygenic architecture with significant environmental influences.

Primary risk factors for complex polygenic conditions like BD and ASD are usually common variants with small effect sizes or rare variants with high penetrance in specific contexts (e.g., CNVs, de novo mutations). While DOCK4 (rs147636134), SYNGAP1 (rs199759879), and FOXP1 (rs767001715) are all genes involved in neurodevelopmental processes and have been implicated in psychiatric and neurodevelopmental disorders. These three SNPs are low‐frequency or ultra‐rare, and studies that report them often suffer from low statistical power, lack of replication, and lack of strong evidence of association with disease in large‐scale genome‐wide association studies (GWAS).

### 4.2 FOXP1: A Key Player in Neurodevelopment

Heterozygous deletions, point mutations, and duplications in *FOXP1* have been linked to ASD, often accompanied by intellectual disability and language impairments (Bacon and Rappold 2012; Hamdan et al. 2010). While *FOXP1* is a high‐confidence ASD risk gene, its association with BD remains tentative, likely due to BD's polygenic nature. Functional studies highlight *FOXP1*’s role in synaptic plasticity, neuronal migration, and corticogenesis (Araujo 2017; Braccioli et al. 2017). Notably, *FOXP1* is highly expressed in the striatum, hippocampus, and neocortex—regions implicated in cognitive and emotional regulation (Ferland et al. 2003). Recurrent *FOXP1* mutations identified in ASD cohorts (Iossifov et al. 2014) underscore its importance in neurodevelopmental pathways.

### 4.3 SYNGAP1: Synaptic Dysfunction in ASD

SYNGAP1, a synaptic Ras‐GTPase activating protein, is strongly associated with ASD and intellectual disability. Loss‐of‐function mutations disrupt excitatory synapse development, leading to synaptic dysfunction (Zhang et al. 2021). Individuals with *SYNGAP1*‐related disorders often exhibit epilepsy, motor deficits, and pronounced language delays (Bednarczuk et al. 2024; Holder et al. 2019). Although *SYNGAP1*’s role in BD is speculative, its impact on synaptic plasticity suggests a potential, albeit indirect, link to mood dysregulation.

### 4.4 DOCK4: A Candidate Gene With Emerging Evidence

DOCK4, involved in neuronal morphogenesis and synaptic transmission, has been tentatively linked to ASD and other neuropsychiatric conditions. Rare deletions and mutations have been reported in ASD cohorts, though it is not yet a high‐confidence risk gene (Girirajan et al. 2011). Limited evidence connects *DOCK4* to BD, possibly through its role in neuronal connectivity. Intriguingly, *DOCK4* has been implicated in both ASD and dyslexia, suggesting shared genetic pathways in communication disorders (Bourgeron 2015; Pagnamenta et al. 2010).

### 4.5 Study Limitations and Future Directions

This study is an important first step in understanding the genetic structure of ASD and BD in Turkiye. Our real‐time PCR analysis of *FOXP1*, *SYNGAP1*, and *DOCK4* SNPs revealed a homozygous genotype in all participants. Global data from gnomAD (2024) show that the minor allele (A) frequencies for these SNPs are extremely low (e.g., *FOXP1* rs767001715: A = 0.00021), which may explain the lack of variation in our cohort.

The SNPs may hint at interesting directions for future exploration, especially within rare variant frameworks or in the context of multi‐omics studies, but as of now, they do not qualify as primary risk factors for either BD or ASD.

#### 4.5.1 Conclusion

In this study, FOXP1 (rs767001715)**, **SYNGAP1 (rs199759879), and DOCK4 (rs147636134) polymorphisms did not demonstrate a significant association with ASD or BD in the Turkish population. However, these findings are preliminary due to the small sample size and restricted SNP selection. Future research with expanded cohorts, whole‐exome sequencing, and functional assays will be essential to clarify the roles of these genes in ASD and BD. Such advances may ultimately inform personalized diagnostic tools and therapeutic strategies for these complex disorders.

Replication in larger, diverse samples and integration with functional genomics data (e.g., Genotype‐Tissue Expression (GTEx), PsychENCODE) would be necessary for future work to be impactful. These kinds of studies contribute to more individualized treatment approaches by offering a crucial basis for comprehending the function of genetic biomarkers in psychiatric disorders.

## Author Contributions

Elvan Çiftçi, Nimet Sağlam, Tayfun Gözler, İpek Yüksel, İlknur Bozkurt, Muhsin Konuk, Korkut Ulucan, Nevzat Tarhan: Conceptualization. Tayfun Gözler, İpek Yüksel, Nimet Sağlam, NK: Data curation. Tayfun Gözler: Formal analysis. Elvan Çiftçi, Nimet Sağlam, Tayfun Gözler, İpek Yüksel, İlknur Bozkurt, Muhsin Konuk, Korkut Ulucan, Nevzat Tarhan: Investigation. Nimet Sağlam, Tayfun Gözler, İpek Yüksel: Methodology. Elvan Çiftçi, Tayfun Gözler: Project administration. Muhsin Konuk, Korkut Ulucan, Nevzat Tarhan: Supervision. Tayfun Gözler, İpek Yüksel: Visualization. Elvan Çiftçi, Tayfun Gözler, İpek Yüksel: Writing original draft preparation. Elvan Çiftçi, Tayfun Gözler, İpek Yüksel, Korkut Ulucan, Muhsin Konuk, Nevzat Tarhan: Writing–review and editing.

## Conflicts of Interest

The authors declare no conflicts of interest.

## Data Availability

Data are shared within the manuscript.
